# Multi-Site Implementation of AI Ambient Voice Technology in Mental Health and Neurodevelopmental Settings: Results From Phase 2

**DOI:** 10.1192/bjo.2026.11491

**Published:** 2026-06-30

**Authors:** Noah Stanton, Brandon Wong, Moustafa Okda, Aadam Aziz, Declan Brogan

**Affiliations:** CNWL, London, United Kingdom

## Abstract

**Aims:**

Child and adolescent mental health services (CAMHS) and child development clinics (CDCs) in the UK are facing unprecedented demand, resulting in prolonged waiting times for assessment of neurodevelopmental conditions, including autism spectrum disorder andattention deficit hyperactivity disorder. Delayed diagnosis and intervention are associated with poorer outcomes, with administrative burden being a key contributor to limited assessment capacity. Emerging digital health technologies like ambient voice technology (AVT) can reduce this burden and improve service efficiency. This second-phase study builds upon the authors’ previous AVT proof-of-concept pilot.

The objectives were to:

1. Assess the performance of AVT across a broad range of use cases in CAMHS outpatient clinics and a CDC.

2. Assess the impact of AVT on quantitative and qualitative clinical outcomes, including administrative burden.

3. Explore clinician, patient and carer perceptions towards AVT in clinical settings.

**Methods:**

We conducted a mixed-methods pre-post service development pilot from January to July 2025. The study was conducted across three outpatient sites: two CAMHS outpatient clinics and one CDC. Clinicians spanning CAMHS and neurodevelopmental roles were invited to participate, testing 13 use cases, mostly diagnostic assessment components for neurodevelopmental conditions. Clinician participants completed both baseline (manual documentation) and intervention (AVT-assisted documentation) phases. The primary outcome measure was self-reported time taken to complete administrative tasks per clinical encounter. Secondary outcome measures included qualitative clinician experience and patient/carer perception and acceptability of AVT. Data was analysed using descriptive statistics and mixed linear regression.

**Results:**

37 clinician participants provided baseline and intervention timesheet data. Mostwere full-time working mental health nurses, aged 25–34 and female. A total of 1,085 clinical appointments were recorded, with 50% (n=543) using AVT. Across all use cases, the mean time taken for administrative tasks was 28% less in the AVT group compared to manual documentation (p<0.001). However, satisfaction with documentation accuracy and confidence in documentation quality were significantly higher with manual documentation (93% and 100%, respectively) compared with AVT (60% and 56%). No significant differences were found in perceived efficiency of seeing patients or completing administrative work. Data from patient/carer surveys revealed no significant differences between AVT and manual documentation across any measures.

**Conclusion:**

AVT significantly reduces documentation burden in neurodevelopmental assessments and is acceptable for patients and carers. However challenges including documentation accuracy and quality must be addressed. Further evaluation across other mental health and neurodevelopmental settings is necessary to validate these findings.

